# Determinants of Postnatal Care Service Utilization Among Mothers in Bangladesh: A Two‐Level Binary Logistic Regression Approach

**DOI:** 10.1002/hsr2.73098

**Published:** 2026-08-26

**Authors:** Md. Obaydul Hossain, Susmita Rani Dey, Uttam Kumar Majumder

**Affiliations:** ^1^ Statistics Discipline Khulna University School of Science Engineering and Technology Khulna Bangladesh

**Keywords:** individual and community level, mothers in Bangladesh, PNC utilization, two‐level binary logistic regression

## Abstract

**Background and Aims:**

Postnatal care is essential for mothers and their children to address any future problems and provide the mothers crucial health information. Most maternal and newborn deaths occur during postnatal period. Maternal mortality can be significantly reduced by increasing the utilization of postnatal care service. This study aimed to identify the prevalence and factors associated with postnatal care (PNC) service utilization among mothers in Bangladesh.

**Methods:**

This study utilized the latest data from the Bangladesh Demographic and Health Survey 2022. A total of 4899 mothers were included for the analysis. Two level binary logistic regression was used to identify associated factors with PNC utilization.

**Results:**

Around 61.6% of mothers utilized PNC service from a medically trained provider within 42 days after delivery. The prevalence was higher among women in Khulna division (79.1%) and lowest was (48.9%) in Sylhet division. In this study, among individual level variables, respondent education, wealth index, place of delivery, mode of delivery and Antenatal care (ANC) visits were significantly associated with PNC utilization. At the community level, community ANC visits and division had a significant association on the utilization of PNC services. Among the significant individual‐level factors, it was found that the place of delivery and the mode of delivery exhibited considerable randomness across different communities.

**Conclusion:**

PNC utilization was significantly associated both individual‐ and community‐level variables. Targeted strategies should be developed to improve PNC utilization, especially in administrative divisions with lower education levels. It is crucial to ensure that women receive at least eight antenatal care visits, and those delivering at home should be strongly encouraged to seek PNC service to improve maternal and child health outcomes.

## 1 Introduction

Postnatal care is essential for mothers and their children to address any future problems and provide the mothers crucial health information. The postnatal period is defined as the phase that starts 1 h after childbirth and lasts up to 6 weeks (42 days) postpartum [1]. A significant number of maternal and newborn deaths take place during this period. The period just after delivery presents significant risks to the mother, including hemorrhage and infection, while the newborn is at higher risk of hypoxia, prematurity, and severe infections. The World Health Organization recommends that all women and babies receive postnatal care from a qualified healthcare professional within 24 h of birth, and attend a minimum of three postnatal checkups within 42 days [2].

Worldwide, around 295,000 maternal deaths result from problems connected to pregnancy and delivery, mostly in developing countries [3]. A recent survey found that only 45% of mothers in countries with low and middle incomes use postnatal care [4], which presents a significant challenge for these countries due to the underutilization of postnatal care. The leading causes of maternal death are unsafe abortions, lengthy labor, postpartum infections, and severe bleeding [5]. In addition, hypertensive disorders of pregnancy such as chronic hypertension, gestational hypertension, preeclampsia, and eclampsia are significant contributors to maternal and neonatal mortality [6]. By 2030, the global community aims to reduce maternal mortality to less than 70 deaths for every 100,000 live births, as part of the third goal of the Sustainable Development Goals (SDG‐3.1) [3, 7].

Bangladesh is a developing country with a significantly high maternal mortality rate. According to the report in a study [8], the maternal mortality rate was 156 deaths per 100,000 live births Bangladesh. Worldwide, about 30% of mother's deaths occurred during the postpartum period. In Bangladesh, this percentage is much higher at 68% [9]. Annually, around 6000 women in Bangladesh lose their lives as a result of inadequate resources in maternity healthcare service [10]. Although 66.5% of Bangladesh's population lives in rural areas and only 44.6% receive facility‐based delivery [11]. In Bangladesh, 52.5% of women received postnatal care service from medically qualified providers within 42 days, as reported by the 2017–2018 BDHS survey [12]. The government of Bangladesh aims to reach 80% of women by 2025 who receive postnatal care from medically qualified professionals within 48 h, and 100% by 2030 [13]. Studies have shown that postnatal care (PNC) service utilization is significantly associated with factors such as place of delivery, decision‐making autonomy, distance to health facilities, number of living children, socioeconomic status, birth order, residence, employment, ethnicity, number of pregnancies, and the husband's occupation [7].

The postnatal period is a vital time for specialized care focused on improving the health and safety of mothers and infants, as many mothers face life‐threatening risks during this phase. Postnatal care is crucial for maintaining the health of both mothers and the child [14]. Postnatal care ensures the detection and management of health conditions in mothers and newborns, including providing iron and vitamin A supplements to support their early well‐being [15]. A study in Bangladesh revealed that women face unequal access to medical facilities within the healthcare system [16]. Additionally, the study found socioeconomic inequality to be one of the primary causes of decreased use of service related to maternal health [17].

In Bangladesh, most research has often neglected the impact of community‐level factors, such as community wealth, Community ANC visits and community media exposure, on postnatal care utilization. However, research from other countries has shown a significant connection between community characteristics and the utilization of PNC [18]. Studies have shown that individual‐level variables alone are insufficient to improve mother's health behaviors; community‐level factors are also necessary [19]. The study aims to estimate the prevalence of PNC utilization among women and to identify individual‐level factors and community‐level factors associated with PNC utilization in Bangladesh.

The outcomes of this study can be used by policymakers to implement effective solutions at the individual and community levels in order to achieve the third Sustainable Development Goal (SDG‐3) and other targets set by the Bangladeshi government to reduce maternal mortality.

## 2 Materials and Methods

### 2.1 Source of Data and Study Design

This study used secondary data from the 2022 Bangladesh Demographic and Health Survey (BDHS), a nationally representative sample of women aged 15–49, to examine the prevalence and associated factors of postnatal care utilization. A stratified household sample was obtained by a two‐stage process. Initially, 675 Enumeration Area (EAs) were chosen, comprising 237 in metropolitan regions and 438 in rural regions, with selection probability proportional to the size of the EA. A systematic sample of, on average, 45 households per EA was picked in the second round of sampling. A total of 30,078 women aged 15–49 who had previously been married were included in the interviews.

From a total of 30,078 responses, we initially selected a sample of 4963 (25,115 women were excluded because they did not have the most recent birth) mothers who had given live birth within 3 years preceding the survey. Women who experienced stillbirths or IUDs were excluded because the study focused on postnatal care utilization following live births. After removing incomplete data, the analysis included 4899 women who had a live birth within 1 h to 42 days postpartum and received postnatal care (PNC) services from medically trained providers, across 675 clusters representing community‐level characteristics.

### 2.2 Variable Declaration

For the purpose of this study, one dependent variable was included. We have chosen eighteen independent variables (thirteen at the individual level and five at the community level) to explain postnatal care utilization.

### 2.3 Dependent Variable

The dependent variable was “postnatal care utilization,” a binary variable with two categories: “Yes” (coded as 1) if the mother received postnatal care from a medically trained provider within 42 days postpartum, and “No” (coded as 0) otherwise. Medically trained providers included qualified doctors, nurses, family welfare visitors, community skilled birth attendants, and sub‐assistant community medical officers [20].

### 2.4 Independent Variables

Several independent variables were included to identify the associated factors of postnatal care (PNC) utilization among mothers in Bangladesh. As this study primarily relied on two‐level analysis, variables from different levels (individual and community) were incorporated accordingly.

This study include thirteen individual‐level factors: respondent's age, religion, birth order, respondent's education, husband's education, respondent's occupation, husband's occupation, media exposure, wealth index, place of delivery, mode of delivery, ANC visits and perceived difficulty of distance to health facility [7, 20].

The study included five community‐level factors: place of residence, division, community wealth, community ANC visits, and media exposure. Place of residence was categorized as “urban” or “rural,” indicating each respondent belonged to one of these community types. The BDHS 2022 dataset includes eight divisions: Barisal, Chittagong, Dhaka, Khulna, Mymensingh, Rajshahi, Rangpur, and Sylhet. The community wealth variable was classified into two categories based on simple ratios. “Poor” for average values below 50% and “Rich” for average values 50% or above. Women who attended less than four ANC visits (< 4 = < 50%) were categorized separately from those who attended four or more visits (≥ 4 = ≥ 50%), defining the community ANC visits variable. Communities with a media exposure average below 50% were classified as “Not exposed,” while those with 50% or more were classified as ‘Exposed [1, 21].

### 2.5 Statistical Analysis

Univariate analysis was used to measure frequency distributions of all individual and community variables. The chi‐square test was also applied to assess the association between the predictors and the dependent variable. The two‐level binary logistic regression analysis included models with fixed, random, and mixed effects associated at both the individual and community levels. Model 1, the null model, did not include any individual‐ or community‐level characteristics. Model 2 included only the variables at the individual level, while Model 3 focused only on the community‐level variables. Finally, the complete model (Model 4) was developed by incorporating the significant community‐level and individual‐level variables identified in Models 2 and 3, respectively.

The two‐level random intercept binary logistic regression model, which accounts for women at level 1 and communities at level 2, can be expressed as follows:

$$\text{logit}\;\left(\pi_{\text{ij}}\right)=\log\left(\frac{\pi_{\text{ij}}}{1-\pi_{\text{ij}}}\right)=\beta_{0j}+\sum_{k=1}^{m}\beta_{k}X_{\text{ijk}};\;i=1,\;2\ldots ,n_{j};j=1,\;2\ldots ,d$$

$$\text{With}\;\beta_{0j}=\;\beta_{0}+\mu_{0j};\;\mu_{0j}\sim \mathrm{iid N}\left(0,\sigma_{\mu 0}^{2}\right)$$

where $\pi_{\text{ij}}=\text{Pr}(Y_{\text{ij}}=1)$, women *i* is community *j* encountered as PNC while giving first birth. $X_{\text{ijk}}$ is the values change by m predictors for women *i* in community *j*. Two‐level model can be applied if value of ICC is greater than zero [22] and also 0.05 [23]. The Intra‐Class Correlation (ICC) represents the ratio of variation at the community level to the total variance. It is an essential tool for assessing the degree of homogeneity within groups, such as families or communities. To calculate the ICC, we employed the linear threshold model approach, which converts individual‐level variation from the probability scale to the logistic scale. This method is used to express community‐level variance, especially when the outcome variable is binary. The presence of multicollinearity among the individual and community‐level components was assessed using the variance inflation factor (VIF). To evaluate best‐fitting modelwe used AIC, BIC and log likelihood. All analysis were conducted using STATA‐17 and IBM SPSS‐26.

## 3 Results

### 3.1 Characteristics of Participants

Table 1 provides a comprehensive overview of the respondent's individual and community level characteristics. Among the 4899 respondents, 3018 (61.6%) received PNC service from a medically trained provider, while 1881 (38.4%) did not receive. The age distribution of respondents included, 2238 (45.7%) women were aged 15–24, 2235 (45.6%) were aged 25–34, and 426 (8.7%) were aged 35–49. The number of Muslim women was 4486 (91.6%), and the rest were Hindu or Christian (categorized as “others”). In terms of birth order, 1816 (37.1%), 2608 (53.2%), 423 (8.4%) and 52 (1.1%) were from women's birth order respectively first, second to third, fourth to fifth and sixth or above. Among respondents, 256 (5.2%) women had no formal education, meanwhile 1125 (22.9%), 2582 (52.7%) and 936 (19.1%) attained primary, secondary and higher education respectively. On the other hand, 1457 (29.7%) of the respondents’ husbands had qualified primary education, 1697 (34.6%) had secondary education, 1012 (20.7%) had higher education, and 256 (5.2%) had no academic education. The most of the women 3664 (74.8%) were homemaker, while a small portion 419 (8.5%) women had professional employment. In terms of husband's occupation,114(2.3%) did not engage any type of work, while 3869 (78.9%) had professional employment. Most of the women were 2778 (56.7%) exposed to the media. Among the respondents, 1002 (20.5%) belong to the poorest wealth, 996 (20.3%) to the poorer wealth, and 978 (19.9%) to the middle, 968 (19.8%), and 955 (19.5%) belong to the richer, and richest wealth.

**Table 1:** Percentage distribution showing the concentration of dependent and independent variables.

Variables	Category	Unweighted frequency, *n* (%)	Weighted frequency, *n* (%)
Total		4899 (100%)	4949 (100%)
**PNC utilization**	Yes	3018 (61.6)	3038 (61.4)
	No	1881 (38.4)	1911 (38.6)
**Respondent age**	15–24	2238 (45.7)	2321 (46.9)
	25–34	2235 (45.6)	2206 (44.6)
	35–49	426 (8.7)	422 (8.5)
**Religion**	Muslim	4486 (91.6)	4576 (92.5)
	Others	413 (8.4)	373 (7.5)
**Birth order**	First	1816 (37.1)	1857 (37.5)
	Second–Third	2608 (53.2)	2615 (52.9)
	Fourth^_^Fifth	423 (8.6)	426 (8.6)
	6th or more	52 (1.1)	51 (1.0)
**Respondent's education**	No education	256 (5.2)	265 (5.3)
	Primary	1125 (22.9)	1107 (22.4)
	Secondary	2582 (52.8)	2690 (54.4)
	Higher	936 (19.1)	887 (17.9)
**Husband's education**	No education	733 (14.9)	757 (15.3)
	Primary	1457 (29.8)	1464 (29.6)
	Secondary	1697 (34.6)	1759 (35.5)
	Higher	1012 (20.7)	969 (19.6)
**Respondent's occupation**	Homemaker	3664 (74.8)	3649 (73.7)
	Agricultural related employment	816 (16.7)	855 (17.3)
	Professional employment	419 (8.5)	445 (9.0)
**Husband's occupation**	Not working	114 (2.3)	117(2.4)
	Agricultural related employment	916 (18.8)	942 (19.0)
	Professional employment	3869 (78.9)	3890 (78.6)
**Media Exposure**	No	2121 (43.3)	2136 (43.2)
	Yes	2778 (56.7)	2813 (56.8)
**Wealth index**	Poorest	1002 (20.5)	990 (20.0)
	Poorer	996 (20.3)	1040 (21.0)
	Middle	978 (19.9)	1025 (20.7)
	Richer	968 (19.8)	985 (19.9)
	Richest	955 (19.5)	909 (18.4)
**Mode of delivery**	Normal delivery	2670 (54.5)	2720 (55.0)
	Cesarean section	2229 (45.5)	2229 (45.0)
**ANC visits**	No visit	385 (7.9)	388 (7.8)
	1–7	4270 (87.2)	4312 (87.1)
	8+	244 (4.9)	249 (5.1)
**Perceived difficulty of distance to health facility**	Big Problem	2179 (44.5)	2230 (45.0)
	Not a big problem	2720 (55.5)	2719 (55.0)
**Place of residence**	Urban	1611 (32.9)	1324 (26.8)
	Rural	3288 (67.1)	3625 (73.2)
**Community wealth**	Poor	1696 (34.6)	1738 (35.1)
	Rich	3203 (65.4)	3211 (64.9)
**Community ANC visits**	< 4	2984 (60.9)	3055 (61.7)
	≥ 4	1915 (39.1)	1894 (38.3)
**Community media exposure**	Not exposed	1706 (34.8)	1669 (33.7)
	Exposed	3193 (65.2)	3280 (66.3)

Among the respondents 2670 (54.5%) had normal delivery, while 2229 (45.5%) had cesarean delivery. The majority of women, 4270 (87.2%), attended !–7 ANC visits, while 244 (4.9%) attended eight or more ANC visits. Over half of the women 2720 (55.5%) reported no major difficulties in accessing healthcare facilities. However, a significant number 2179 (44.5%) had faced problems. Most of the respondents lived in rural area 3288 (67.1%) while the remaining 1611 (32.9%) women lived in urban area. The majority women belong to rich community wealth 3203 (65.4%) while 1696 (34.6%) belong to the poor community. In terms of community ANC visits, 1915 (39.1%) attended four or more antenatal care visits, while 2984 (60.9%) attended less than four visits. Among the respondents, 3193 (65.2%) women were exposed to the media at the community level.

**Figure 1:**
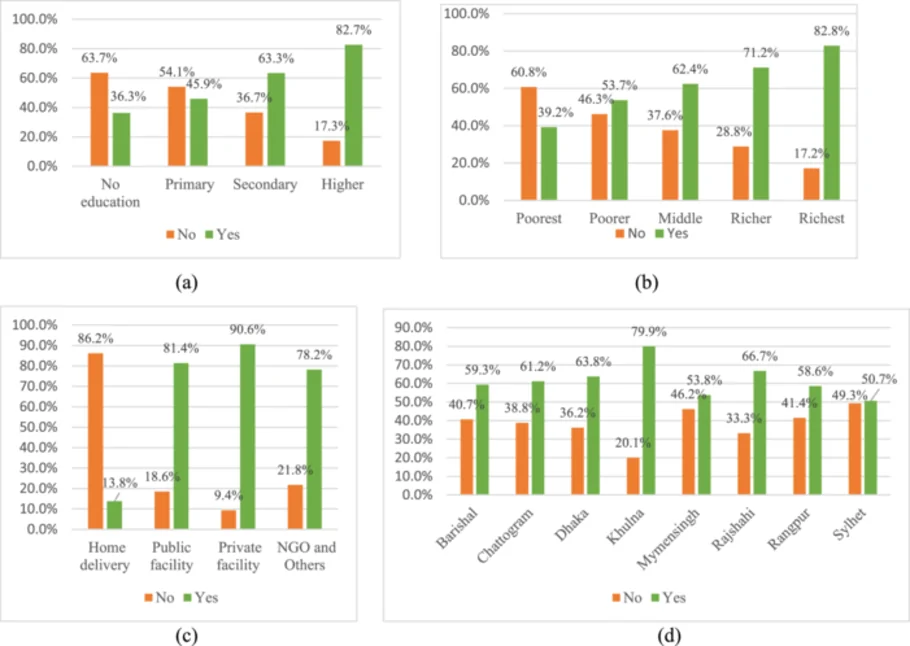
Prevalence of PNC utilization among women across Respondent's education (a), Wealth Index (b), Place of delivery (c) and Division (d).

From Figure 1, Mothers with higher education had the highest prevalence (82.7%) of receiving postnatal care from a medically trained provider compared with mothers who had no education, primary education, or secondary education. Additionally, the highest prevalence was observed in the richest wealth index group (82.8%), private facility delivery (90.6%), and the Khulna division (79.9%).

### 3.2 Prevalence of PNC Utilization

Table 2 presents the association between PNC utilization of mothers and various independent variables at both individual levels and community levels, analyzed using the chi‐square (χ^2^) test, with corresponding p‐values. All independent variables in this study showed a significant association with the dependent variable (PNC utilization). Additionally, bivariate analysis was performed to evaluate the weighted prevalence of PNC utilization across various respondent groups.

**Table 2:** Association between PNC utilization of mothers and different independent variables.

Factors		PNC utilization		*p* value	Weighted prevalence Yes, *n* (%)
		Yes, *n* (%)	No, *n* (%)		
**Respondent age**	15–24	1414 (63.18)	824 (36.82)	< 0.05*	1464 (63.09)
	25–34	1364 (61.03)	871 (38.97)		1337 (60.62)
	35–49	240 (56.36)	186 (43.66)		237 (56.03)
**Religion**	Muslim	2703 (60.25)	1783 (39.75)	< 0.001**	2759 (60.31)
	Others	315 (76.27)	98 (23.73)		279 (74.66)
**Birth order**	First	1251 (68.89)	565 (31.11)	< 0.001**	1263 (68.06)
	Second–Third	1575 (60.39)	1033 (39.61)		1577 (60.31)
	Fourth^_^Fifth	179 (42.32)	244 (57.68)		186 (43.58)
	Sixth or more	13 (25.00)	39 (75.00)		12 (23.06)
**Respondent's education**	No education	93 (36.33)	163 (63.67)	< 0.001**	101 (38.03)
	Primary	516 (45.87)	609 (54.13)		504 (45.54)
	Secondary	1635 (63.35)	947 (36.68)		1702 (63.25)
	Higher	774 (82.69)	162 (17.31)		731 (82.50)
**Husband's education**	No education	308 (42.02)	425 (57.98)	< 0.001**	321 (42.34)
	Primary	743 (51.00)	714 (49.00)		751 (51.29)
	Secondary	1136 (66.94)	561 (33.06)		1163 (66.17)
	Higher	831 (82.11)	181 (17.89)		803 (82.85)
**Respondent's occupation**	Homemaker	2294 (62.61)	1370 (37.39)	< 0.001**	2284 (62.60)
	Agricultural related employment	445 (54.53)	371 (45.47)		461(53.91)
	Professional employment	279 (66.69)	140 (33.41)		293(65.81)
**Husband's occupation**	Not working	70 (61.40)	44 (38.60)	< 0.001**	70(60.00)
	Agricultural related employment	437(47.71)	479 (52.29)		459(48.69)
	Professional employment	2511(64.90)	1358 (35.10)		2509(64.50)
**Media Exposure**	No	1094(51.58)	1027 (48.42)	< 0.001**	1104 (51.69)
	Yes	1924(69.26)	854 (30.74)		1934 (68.75)
**Wealth index**	Poorest	393(39.22)	609 (60.78)	< 0.001**	395 (39.86)
	Poorer	535(53.71)	461 (46.29)		564 (54.27)
	Middle	610(62.37)	368 (37.63)		642 (62.60)
	Richer	689(71.18)	279 (28.82)		689 (70.00)
	Richest	791(82.83)	164 (17.17)		748 (82.27)
**Place of delivery**	Home	239(13.80)	1493 (86.20)	< 0.001**	241 (13.71)
	Public facility	684(81.43)	156 (18.57)		684 (81.79)
	Private facility	2009(90.62)	208 (9.38)		2033 (90.36)
	NGO or others	86(78.18)	24 (21.82)		80 (76.09)
**Mode of delivery**	Normal delivery	954(35.73)	1716 (64.27)	< 0.001**	978 (35.94)
	Cesarean section	2064(92.60)	165 (7.40)		2060 (92.44)
**ANC visits**	No visit	83(21.56)	302 (78.44)	< 0.001**	88 (22.79)
	1–7	2723 (63.77)	1547 (36.23)		2730 (63.30)
	8+	212 (86.89)	32 (13.11)		220 (88.35)
**Perceived difficulty of distance to health facility**	Big Problem	1264 (58.01)	915 (41.99)	< 0.001**	1309 (58.68)
	Not a big problem	1754 (64.49)	966 (35.51)		1729 (63.61)
**Place of residence**	Urban	1143 (70.95)	468 (29.05)	< 0.001**	927 (70.00)
	Rural	1875 (57.03)	1413 (42.97)		2111 (58.24)
**Division**	Barisal	318 (59.33)	218 (40.67)	< 0.001**	168 (54.18)
	Chattogram	517 (61.18)	328 (38.82)		642 (59.01)
	Dhaka	463 (63.77)	263 (36.23)		781 (64.54)
	Khulna	442 (79.93)	111 (20.07)		404 (79.09)
	Mymensingh	326 (53.80)	280 (46.20)		228 (52.36)
	Rajshahi	331 (66.73)	165 (33.27)		344 (65.92)
	Rangpur	332 (58.55)	235 (41.45)		314 (56.88)
	Sylhet	289 (50.70)	281 (49.30)		157 (48.94)
**Community wealth**	Poor	786 (46.34)	910 (53.66)	< 0.001**	817 (47.01)
	Rich	2232 (69.68)	971 (30.32)		2221 (69.17)
**Community ANC visits**	< 4	1594 (53.42)	1390 (46.58)	< 0.001**	1647 (53.92)
	≥ 4	1424 (74.36)	491 (25.64)		1391 (73.44)
**Community media exposure**	Not exposed	838 (49.12)	868 (50.88)	< 0.001**	820 (49.14)
	Exposed	2180 (68.27)	1013 (31.73)		2218 (67.62)

*significant at *p *< 0.05***significant at *p *< 0.001

Respondents aged 15–24 years exhibited the highest PNC utilization rate at (63.09%), while those aged 35–49 years recorded the lowest rate at (56.03%). Higher utilization rates were observed among non‐Muslim respondents (74.66%), those with a first‐born child (68.06%), women with higher education (82.50%), and those whose husbands had higher education (82.85%). Additionally, women engaged in professional employment (65.81%), those whose husbands were professionally employed (64.50%), and women with media access (68.75%) showed higher rates of PNC utilization. In contrast, lower utilization rates were noted among Muslim respondents (60.31%), women with a sixth or higher birth order child (23.06%), those with no education (38.03%), and those whose husbands lacked formal education (42.34%). Similarly, women involved in agricultural employment (53.91%), those whose husbands were engaged in agricultural work (48.69%), and those without media exposure (51.69%) demonstrated reduced PNC utilization rates. The study highlighted notably higher PNC utilization among mothers from the richest families (82.27%), those who delivered at private facilities (90.36%), had cesarean deliveries (92.44%), and attended eight or more antenatal care visits (88.35%). Conversely, the lowest utilization rates were observed among mothers from the poorest families (39.86%), those who delivered at home (13.71%), had normal deliveries (35.94%), or did not attend any antenatal care visits (22.79%).

At the community level, PNC utilization was notably higher among mothers residing in urban areas (70.00%), those in the Khulna division (79.09%), and mothers from communities with greater wealth (69.17%). Similarly, higher utilization rates were observed among women exposed to community media (67.62%) and those in communities where four or more ANC visits were attended (73.44%). In contrast, lower PNC utilization rates were recorded among mothers in rural areas (58.24%), the Sylhet division (48.94%), communities with lower wealth (47.01%), those with fewer than four antenatal care visits at the community level (53.92%), and women not exposed to community media (49.14%).

Table 3 presents the variance inflation factor (VIF) values for all independent variables. All VIF values are less than 5, indicating that there is no evidence of multicollinearity among the explanatory variables.

**Table 3:** Multicollinearity assessment using variance inflation factors (VIF).

Variables	VIF	Variables	VIF
Respondent age	1.04	Place of delivery	2.24
Religion	1.03	Mode of delivery	2.12
Birth order	1.73	ANC visits	1.17
Respondent's education	1.67	Perceived difficulty of distance to health facility	1.02
Husband's education	1.68	Place of residence	1.31
Respondent's occupation	1.04	Division	1.04
Husband's occupation	1.07	Community wealth	1.52
Media Exposure	1.38	Community ANC visits	1.25
Wealth index	1.95	Community media exposure	1.48

### 3.3 Two‐Level Binary Logistic Regression Analysis

Table 4 displays the results of the two‐level binary logistic regression analysis for the outcome variable PNC utilization, considering both individual levels and community‐levels predictors. Model 1, also referred to as the Null Model or intercept‐only model, was designed to evaluate community‐level variation. The intra‐class correlation coefficient (ICC) for this null model is 20.91%, which is greater than0.05 [23] indicates meaningful clustering effects within communities and supports the use of a multilevel modeling approach. This signifies that 20.91% of the overall variance in PNC service is attributed to variations among communities. The proportional change in variance (PCV), relative to the null model, was 32.22% in Model 2, 62.06% in Model 3, and 37.93% in Model 4, indicating reductions in the unexplained community‐level variance after inclusion of the respective individual‐ and community‐level covariates. Model 4 had the lowest AIC (3527.29), BIC (3702.71), and the highest log‐likelihood (−1736.64) among the four models, indicating the overall final fitting model. Therefore, Model 4 was selected as the final model for interpretation.

**Table 4:** Two‐level binary logistic regression analysis of individual and community level factors associated with PNC utilization in Bangladesh.

Factors	Model 1 (Null model)	Model 2 (Individual level)		Model 3 (Community level)		Model 4 (Individual and community level)	
		AOR (95% CI)	*p* value	AOR (95% CI)	*p* value	AOR (95% CI)	*p* value
**Individual level factors**							
**Respondent age**							
15–24		Ref					
25–34		0.91 (0.72–1.16)	0.463				
35–49		1.29 (0.85–1.95)	0.233				
**Religion**							
Muslim		Ref					
Others		1.09 (0.75–1.58)	0.656				
**Birth order**							
1st		Ref					
2^nd_^3rd		0.95 (0.75–1.91)	0.707				
4^th_^5th		0.91 (0.60–1.38)	0.651				
6th or more		0.81 (0.30–2.19)	0.671				
**Respondent's education**							
No education		Ref				Ref	
Primary		1.38 (0.87–2.17)	0.169			1.42 (0.91–2.20)	0.119
Secondary		1.54 (0.97–2.42)	0.065			1.62 (1.06–2.48)	**0.027** *
Higher		1.77 (1.03–3.04)	**0.037** *			1.91 (1.17–3.11)	**0.010** *
**Husband's education**							
No education		Ref					
Primary		1.16 (0.87–1.56)	0.310				
Secondary		1.26 (0.93–1.70)	0.143				
Higher		1.25 (0.84–1.85)	0.264				
**Respondent's occupation**							
Homemaker		Ref				Ref	
Agricultural related employment		1.30 (1.01–1.69)	**0.044** *			1.28 (0.99–1.66)	0.056
Professional employment		1.20 (0.84–1.70)	0.310			1.23 (0.87–1.75)	0.243
**Husband's occupation**							
Not working		Ref					
Agricultural related employment		1.39 (0.75–2.57)	0.290				
Professional employment		1.66 (0.94–2.95)	0.082				
**Media exposure**							
No		Ref					
Yes		0.86 (0.70–1.05)	0.147				
**Wealth index**							
Poorest		Ref				Ref	
Poorer		1.23 (0.92–1.63)	0.161			1.22 (0.92–1.62)	0.158
Middle		1.31 (0.97–1.76)	0.079			1.33 (0.98–1.80)	0.069
Richer		1.38 (1.00–1.90)	**0.049** *			1.39 (1.00–1.92)	**0.048** *
Richest		1.57 (1.08–2.27)	**0.017** *			1.52 (1.05–2.21)	**0.026** *
**Place of delivery**							
Public facility		Ref				Ref	
Home		0.042 (0.03–0.06)	**< 0.001** ***			0.04 (0.03–0.06)	**< 0.001** ***
Private facility		1.13 (0.85–1.51)	0.396			1.13 (0.85–1.50)	0.403
NGO or others		0.74 (0.42–1.29)	0.288			0.72 (0.41–1.25)	0.243
**Mode of delivery**							
Normal delivery		Ref				Ref	
Cesarean section		3.72 (2.81–4.92)	**< 0.001** ***			3.85 (2.91–5.09)	**< 0.001** ***
**ANC visits**							
No visit		Ref				Ref	
1−7		1.65 (1.14–2.39)	**0.008** **			1.68 (1.17–2.43)	**0.005** *
8+		2.23 (1.19–4.16)	**0.012** *			2.23 (1.19–4.17)	**0.012** *
**Perceived difficulty of distance to health facility**							
Big Problem		Ref					
Not a big problem		1.04 (0.86–1.25)	0.691				
**Community level factors**							
**Place of residence**							
Urban				Ref			
Rural				0.87 (0.72–1.05)	0.145	—	—
**Division**							
Khulna				Ref		Ref	
Barisal				0.56 (0.39–0.80)	**0.002** **	0.86 (0.53–1.41)	0.560
Chattogram				0.46 (0.33–0.64)	**< 0.001** ***	1.04 (0.67–1.61)	0.875
Dhaka				0.39 (0.28–0.54)	**< 0.001** ***	0.52 (0.34–0.82)	**0.005** ***
Mymensingh				0.40 (0.28–0.56)	**< 0.001** ***	0.71 (0.44–1.15)	0.169
Rajshahi				0.48 (0.34–0.68)	**< 0.001** ***	0.56 (0.35–0.91)	**0.020** *
Rangpur				0.47 (0.33–0.66)	**< 0.001** ***	0.63 (0.39–1.01)	0.053
Sylhet				0.34 (0.24–0.48)	**< 0.001** ***	0.70 (0.44–1.13)	0.143
**Community wealth**							
Poor				Ref		Ref	
Rich				1.88 (1.56–2.28)	**< 0.001** ***	0.94 (0.71–1.25)	0.682
**Community ANC visits**							
< 4				Ref		Ref	
≥ 4				1.87 (1.57–2.23)	**< 0.001** ***	1.30 (1.02–1.66)	**0.032** *
**Community media exposure**							
Not exposed				Ref		Ref	
Exposed				1.48 (1.23–1.79)	**< 0.001** ***	0.95 (0.73–1.23)	0.677
**Random effect**	**Model 1 (null model)**	**Model 2 (Individual level)**		**Model 3 (community level)**		**Model 4 (Individual and Community level)**	
Community variance (SE)	0.87*(0.10)	0.59*(0.12)		0.33*(0.06)		0.54*(0.12)	
ICC (%)	20.91	15.21		9.11		14.18	
${\mathrm{PCV}}^{i}$ (%)	Ref	32.22		62.06		37.93	
**Model fitness**							
Log Likelihood	−3125.86	−1742.31		−2996.51		−1736.64	
AIC	6255.73	3544.62		6019.02		3527.29	
BIC	6268.73	3739.52		6103.48		3702.71	

*Note:* Significant at probability level.

Compared with the intercept or null model.

* *p* < 0.05

** *p* < 0.01

*** *p* < 0.001.

### 3.4 Individual Level Factors

In the two‐level binary regression analysis, Model 4 revealed that respondent education, wealth index, place of delivery, mode of delivery, and ANC visits were significant factors associated with the utilization of PNC service. Women who had completed secondary education were 1.62 times more likely (AOR: 1.62, 95% CI: 1.06‐2.48) and those with higher education were 1.91 times more likely (AOR: 1.91, 95% CI: 1.17–3.11) to receive PNC service compared to women with no formal education. Women from wealthier households had a higher likelihood of receiving postnatal care. Specifically, those from richer‐income families were 1.39 times more likely (AOR: 1.39, 95% CI: 1.00–1.92) to utilize PNC compared to women from the poorest households. Similarly, women from the richest families were 1.52 times more likely (AOR: 1.52, 95% CI: 1.05–2.21) to receive PNC service. Mothers who delivered at home had approximately 96% lower odds to receive PNC service compared to those who delivered at a public facility (AOR: 0.04, 95% CI: 0.03‐0.06). Mothers who underwent a cesarean section were 3.85 times more likely (AOR: 3.85, 95% CI: 2.91–5.09) to utilize PNC service compared to those who had a normal delivery. Mothers who attended 1 to 7 antenatal care (ANC) visits were 1.68 times more likely (AOR: 1.68, 95% CI: 1.17–2.43) to receive PNC service compared to those who did not attend any ANC visits. Similarly, mothers who attended eight or more ANC visits were 2.23 times more likely (AOR: 2.23, 95% CI: 1.19–4.17) to receive PNC compared to mothers who did not attend ANC.

### 3.5 Community Level Factors

At the community level, division and community ANC visits were found to have a significant impact on the utilization of postnatal care (PNC) service among mothers. Women living in the Dhaka Division had 44% lower odds (AOR: 0.52, 95% CI: 0.34–0.82) to receive postnatal care services compared to women living in the Khulna Division. Similarly, women from the Rajshahi Division were 0.56 times less likely (AOR: 0.56, 95% CI: 0.35–0.91) to receive postnatal care service from medically trained providers compared to women from the Khulna Division. Women living in communities where four or more antenatal care visits were attended had 1.30 times higher odds (AOR: 1.30, 95% CI: 1.02–1.66) of receiving postnatal care services compared to women in communities with less than four antenatal care visits.

### 3.6 Randomness of Individual‐Level Factors

In this analysis, we assessed the randomness of significant individual‐level predictors using the Likelihood Ratio (LR) test, the results of which are presented in Table 5. Among the significant individual‐level factors identified in Model 4, it was found that both the place of delivery and the mode of delivery exhibited considerable variation across different communities.

**Table 5:** Testing randomness of individual level factors across communities.

Individual factors	Model without random slope ($M_{1}$)	Model with random slope ($M_{2}$)	Likelihood ratio (LR) statistic 2($M_{2}$‐ $M_{1}$)	$\chi_{\left(2,0.05\right)}^{2}$
**Wealth index (Poorest vs.)**	−1748.98			5.99
Poorer		−1748.94	0.08	
Middle		−1746.57	4.82	
Richer		−1748.20	1.56	
Richest		−1748.86	0.24	
**Respondent's education (No education vs.**)				
Primary		−1748.97	0.02	
Secondary		−1748.59	0.78	
Higher		−1746.23	5.5	
**Mode of delivery (Normal vs.)**				
Cesarean		−1740.79	16.38**	
**Place of delivery (Public facility vs.)**				
Home		−1740.53	16.9**	
Private facility		−1748.22	1.52	
Ngo or others		−1747.79	2.38	
**ANC visit (No visit vs.)**				
1–7		−1746.21	5.54	
≥ 8		−1746.41	5.14	

*significant at *p *< 0.05**significant at *p *< 0.01

## 4 Discussion

This study demonstrated that respondent education, wealth index, place of delivery, mode of delivery, ANC visits, division, and community ANC visits were significant associated factors of PNC utilization. The prevalence of PNC utilization among mothers within 42 days from a medically trained provider was 61.60%. This is slightly lower than with other countries, such as India (65%) [24], Indonesia (78.5%) [25] and Zambia (63%) [26]. This study found wealth index (an individual level factor) is an influencing factor for determining postnatal care utilization. Women from richer and richest family were more likely to utilize PNC service compared to the poorest family. Existing research [7, 13, 27] also revealed that mothers from wealthier families had more possibility to utilize PNC services, which is consistent with the findings of this study. A possible explanation is that mothers from wealthier households are more likely to be educated and have greater autonomy in making decisions regarding their own health, which facilitates PNC utilization. ANC visits (an individual level factor) was a significant associated factor for the PNC utilization from medically trained provider among mothers. Previous studies using different statistical method found four and more than four ANC visits lead to higher utilization of PNC in Bangladesh [20, 28] and Kenya [29], which is similar to this study finding. Using multilevel analysis for PNC, Ethiopian [21] and Tanzanian [1] researcher also found same ANC visits associated factors. One reason could be that antenatal care counseling increases mothers’ understanding of the significance and accessibility of PNC services. Mode of delivery is also an important factor to utilize postnatal care services, which is found significant in this study. Some existing research [30, 31] had showed that mothers who deliver via cesarean section were more likely to receive PNC services compared to those who deliver vaginally, which is also similar in this study. This may be because cesarean delivery can raise the risk of postpartum problems and include prolonged hospital stays, which may increase PNC chances. Place of delivery (an individual‐level factor) was found to be significant in this study as a determinant of PNC utilization from a medically trained provider. Women who had delivery at home were less likely to utilize PNC services compared to the mothers who had public facility delivery. Several previous studies [26, 31, 32] also found that women who delivered at a healthcare institution were more likely to utilize PNC service compared to mothers who delivered at home. Economic barriers such as transportation costs and medical expenses were identified as potential obstacles to PNC services. Respondent education also found significant with PNC utilization. Women from secondary and higher education were more likely utilize PNC service compare to women who had no formal education. Which was similar with previous paper [20] and [27]. Educated women are more likely to understand the importance of postnatal care, possess greater health awareness and make informed decisions, which may contribute to higher PNC utilization. Community ANC visits was significantly associated with PNC utilization. Women in the community who received four or more ANC services were significantly more likely to utilize PNC compared to those who received fewer than four ANC services. Very few existing studies tried to associate community ANC visits with utilization of PNC services. A study by [1] attempted to identify a correlation between community ANC visit and PNC utilization, but found no significant association. Another important variable is division, which was found significant in this study. Women from Dhaka division and Rajshahi division were less likely to utilize PNC service than those from Khulna. Studies [13, 20] had shown significant variations in PNC service utilization across different divisions in Bangladesh. Geographic, socioeconomic, and healthcare system differences may contribute to the observed divisional variation in postnatal care utilization.

## 5 Strengths and Limitations

The study used nationally representative BDHS data 2022 from Bangladesh, allowing for generalization to the entire women population. In addition, the two‐level binary logistic regression modeling framework applied for identification of both individual‐ and community‐level factors and assessed the random variations across communities. This two‐level approach provides a clearer understanding of the key determinants compared to single‐level methods. However, several limitations should be acknowledged. First, the analysis relied on secondary data, and the study was consequently restricted to variables available in the BDHS 2022 dataset; some potentially relevant factors may not have been measured or available for analysis. Second, the findings may be subject to recall and reporting biases because some information was self‐reported. Finally, cross‐level interactions could not be examined because of insufficient variability at the community level.

## 6 Conclusion

In conclusion, this study highlighted a statistically significant association among observations within the same cluster. PNC utilization was significantly associated by both individual‐ and community‐level variables. Among the individual‐level variables, respondent education, wealth index, place of delivery, mode of delivery, and ANC visits showed significant associations. Similarly, among the community‐level variables, division and community ANC visits were significantly associated with PNC utilization. Moreover, the effects of mode of delivery and place of delivery on PNC utilization were found to vary randomly across communities. Based on the findings of this study, the following recommendations are proposed: Targeted strategies should be developed to improve PNC utilization, especially in administrative divisions with lower education levels. It is crucial to ensure that women receive at least eight antenatal care visits, and those delivering at home should be strongly encouraged to seek PNC service to improve maternal and child health outcomes. Future studies could explore cross‐level interaction effects by incorporating level‐3 modeling approach and including additional community‐level factors to better explain variations in postnatal care (PNC) utilization.

## Author Contributions

**Md. Obaydul Hossain:** conceptualization, data curation, formal analysis, methodology, resources, software, writing – original draft, writing – review and editing. **Susmita Rani Dey:** formal analysis, methodology, software, writing – original draft, writing – review and editing. **Uttam Kumar Majumder:** conceptualization, formal analysis, supervision, validation, writing – original draft, writing – review and editing.

## Ethics Statement

Ethical approval was not necessary since this study utilized nationally representative secondary data from a reliable and established Bangladesh Demographic and Health Survey (BDHS) source.

## Conflicts of Interest

The authors declare no conflicts of interest.

## Transparency Statement

The lead author Md. Obaydul Hossain affirms that this manuscript is an honest, accurate, and transparent account of the study being reported; that no important aspects of the study have been omitted; and that any discrepancies from the study as planned (and, if relevant, registered) have been explained.

## Data Availability

The data that support the findings of this study are openly available in Bangladesh Demographic and Health Survey (BDHS) at https://dhsprogram.com/data/available-datasets.cfm.
